# National malaria vector control policy: an analysis of the decision to scale-up larviciding in Nigeria

**DOI:** 10.1093/heapol/czv055

**Published:** 2015-06-15

**Authors:** Kemi Tesfazghi, Jenny Hill, Caroline Jones, Hilary Ranson, Eve Worrall

**Affiliations:** ^1^Department of Vector Biology, Liverpool School of Tropical Medicine, Pembroke Place, UK,; ^2^Department of Clinical Sciences, Liverpool School of Tropical Medicine Pembroke Place, UK,; ^3^Department of Disease Control, London School of Hygiene and Tropical Medicine, Keppel Street, UK and; ^4^Department of Public Health Research, KEMRI-Wellcome Trust Research Programme, Kilifi, Kenya

**Keywords:** Larviciding, larval source management, malaria, policy analysis, vector control

## Abstract

**Background:** New vector control tools are needed to combat insecticide resistance and reduce malaria transmission. The World Health Organization (WHO) endorses larviciding as a supplementary vector control intervention using larvicides recommended by the WHO Pesticides Evaluation Scheme (WHOPES). The decision to scale-up larviciding in Nigeria provided an opportunity to investigate the factors influencing policy adoption and assess the role that actors and evidence play in the policymaking process, in order to draw lessons that help accelerate the uptake of new methods for vector control.

**Methods: **A retrospective policy analysis was carried out using in-depth interviews with national level policy stakeholders to establish normative national vector control policy or strategy decision-making processes and compare these with the process that led to the decision to scale-up larviciding. The interviews were transcribed, then coded and analyzed using NVivo10. Data were coded according to pre-defined themes from an analytical policy framework developed *a priori.*

**Results: **Stakeholders reported that the larviciding decision-making process deviated from the normative vector control decision-making process. National malaria policy is normally strongly influenced by WHO recommendations, but the potential of larviciding to contribute to national economic development objectives through larvicide production in Nigeria was cited as a key factor shaping the decision. The larviciding decision involved a restricted range of policy actors, and notably excluded actors that usually play advisory, consultative and evidence generation roles. Powerful actors limited the access of some actors to the policy processes and content. This may have limited the influence of scientific evidence in this policy decision.

**Conclusions: **This study demonstrates that national vector control policy change can be facilitated by linking malaria control objectives to wider socioeconomic considerations and through engaging powerful policy champions to drive policy change and thereby accelerate access to new vector control tools.

Key Messages

- Policy analysis can be used to aid our understanding of how to accelerate policy change. In the field of malaria vector control, policy analysis has so far revealed concerns about donor pressure and lack of engagement of national level politicians. It has highlighted the potential for policy champions, international networks and involvement of researchers in policy development to aid translation of research into policy.
- Additional vector control tools are needed to combat insecticide resistance and reduce malaria transmission. Larviciding is endorsed by the World Health Organization (WHO) as a supplementary vector control intervention that has been adopted by relatively few African endemic countries. The uptake of larviciding by policymakers in West Africa presents an opportunity to better understand policymaking processes for vector control interventions and accelerate access to new vector control tools.
- The larviciding policy process in Nigeria deviated from the normative vector control process. It was initiated at the highest political levels involving a restricted range of actors, notably excluding those that usually play advisory, consultative and evidence generation roles. This may have limited the influence of scientific evidence. The potential of larviciding to contribute to national economic development objectives was cited as a key factor influencing support for this policy.
- Uptake or scale up of malaria control can be facilitated by linking malaria control objectives to wider economic considerations and through engaging powerful policy champions to drive policy change. However, care needs to be taken to ensure that evidence of effectiveness is also central to the policy process.

## Background

The scale-up of vector control has been critical to the reduction in malaria transmission seen over the past decade (World Health Organization 2012b). Key tools for vector control include long lasting insecticide-treated nets (LLINs) and indoor residual spraying (IRS) (World Health Organization 2013d). In sub-Saharan Africa, the percentage of households owning at least one insecticide-treated net increased from 3 to 54% between 2000 and 2013 (World Health Organization 2013d) with the number of nets delivered to malaria endemic countries by manufactures increasing from 6 to 136 million between 2004 and 2013 (World Health Organization 2013d). However, new vector control tools are urgently needed, to combat the increasing resistance that is threatening the effectiveness of existing insecticide-based interventions (Ranson *et al.* 2011; World Health Organization 2012b) and to control malaria vectors not targeted by current interventions (e.g. those that bite outdoors).

Larval source management (LSM) is the management of water bodies that are potential breeding sites for malaria vectors. It includes habitat modification or the addition of chemicals to water bodies to prevent the development of adult mosquitoes (larviciding). Larviciding has been recognized as a valuable addition to malaria vector control in specific settings. WHO recommends that in sub-Saharan Africa, LSM should only be implemented as a supplement to LLINs and IRS in clearly defined habitats, particularly in urban areas where malaria vector breeding sites are few, fixed and findable (World Health Organization 2012a; World Health Organization 2013c). In 2012, national malaria control programmes in six African countries reported using larviciding (World Health Organization 2013c).

In recent years, the Economic Union of West African States (ECOWAS) has generated a renewed interest in scaling-up larviciding in West Africa. A tripartite agreement, between ECOWAS, Venezuela and Cuba was signed in 2009 to provide financial and technical support to scale-up larviciding in the region with a view to eliminating malaria. Technology transfer for the establishment of microbial larvicide factories in Ghana, Nigeria and Cote d’Ivoire forms part of the agreement, in a bid to create jobs and make larvicides readily available in the region (ECOWAS, 2013). Microbial larvicides have been shown to be protective against malaria (Fillinger and Lindsay 2006; Geissbuhler *et al.* 2009), but only one strain (*Bacillus thuringiensis* subsp. israelensis, strain AM65-52, WG) has been approved for larviciding by the WHO’s Pesticide Evaluation Scheme (WHOPES) (World Health Organization 2013b). The ECOWAS larviciding plans involve the use of two larvicides produced by the Cuban company, Labiofam. These larvicides, BACTIVEC (*Bacillus thuringiensis* SH-14) and GRISELESF (*Bacillus sphaericus* stump 2362), do not currently have WHOPES approval.

Malaria is endemic in Nigeria and remains a serious public health problem with 97% of the total population at risk of infection (National Population Commission and National Malaria Control Programme 2010). LLINs are the main prevention strategy in the country with the current National Malaria Strategic Plan (NMSP) aiming for 80% LLIN ownership and use by 2013 (National Malaria Control Programme 2009b). However, only 41% of households have at least one LLIN (National Population Commission and National Malaria Control Programme 2010). IRS is considered a complementary strategy to LLINs in Nigeria and has been piloted in some states (coverage 1% within the targeted states), with the objective of being scaled-up to cover 20% of the targeted states’ population, primarily in urban areas by 2013 (National Population Commission and National Malaria Control Programme 2010). LSM (including larviciding) is included in the current NMSP (National Malaria Control Programme 2009b; World Health Organization 2013a), but its use to date has been extremely limited. Thus, plans to scale-up larviciding nationwide, using non-WHOPES approved products, represents a deviation from the current malaria control strategy in Nigeria.

Given the alarming rise in insecticide resistance in Africa, it is likely that many countries are going to have to consider changing their vector control policy and deploying additional vector control interventions. The decision to scale-up larviciding in Nigeria provided an opportunity to investigate the factors influencing policy adoption and assess the role that actors and evidence play in the policymaking process in order to draw lessons that help accelerate the uptake of new methods for vector control.

## Methods

### Analytical framework

**Figure 1.**
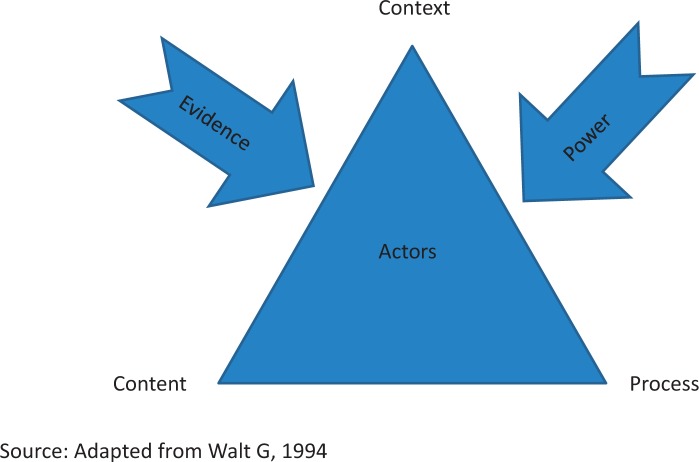
Analytical framework

A review of the literature on policy analysis was carried out to identify suitable analytical frameworks for policy analysis. An analytical framework which combines the policymaking context, actors, process, content, power (Walt 1994) and the role of evidence in policymaking (Court and Young 2006) was developed (Figure 1; Table 1). The framework was used to guide all aspects of the study from the identification of documents for the desk review; identification of key informants (KI), development of study instruments and data analysis. The concept of power, which can be expressed in various ways, is a crucial element of the Walt and Gilson framework (Walt 1994)*.* In this article, we investigate a number of dimensions of power expressed in the policy process including ‘decision-making’ (Dahl, 1961), ‘agenda setting’, (Bachrach and Baratz 1962), ‘thought control’ (Lukes 1974) and the ability to undermine influence (Radcliffe 2000). Recognizing that power is methodologically difficult and sensitive to investigate (Erasmus and Gilson 2008; Lehmann and Gilson 2013) we sought to gather information by asking questions on ‘which actors carried the most influence in the policy process and why’.

**Table 1.** Definitions of terms used in Analytical Framework (Adapted from Walt 1994)

Framework category	Definition
Context	Systemic factors, including political, economic and social, at national and international levels, that influence vector control policy
Actors	Stakeholders (individuals or organizations) that make/influence vector control policy
Process	The way polices are developed
Content	The technical content of the specific policy under analysis
Evidence in Policy Making	‘Any form of knowledge, including, but not confined to research, of sufficient quality to be used to inform decisions’ (Buse *et al.* 2012)
Power	The ability to influence, and in particular, the ability to control resources. Power is characterized by authority, finances and access to knowledge

### Document review

A review of published and unpublished national documents was undertaken to: understand the national vector control policy context; identify the content of the national vector control policies; identify the key actors involved in national vector control and inform the development of the semi-structured interview guide. Documents reviewed included: national malaria policies, strategies and guidelines; national malaria vector control policies, strategies and guidelines; organograms and structures of Federal Ministry of Health (FMOH); terms of reference and minutes of meetings of national policymaking bodies; policies, strategies, action plans, press releases and web pages of policymaking bodies, as well as research, implementing and financing institutions involved in malaria vector control in Nigeria.

Documents were sourced through on-line searches (Google Scholar and PubMed) and requests to relevant individuals and organizations. The review was supplemented with documents identified by stakeholders during interviews.

To guide the document review, different categories of policy (health, meso and macro) and strategy, as used by Mays (Mays 2011) and Buse (2012) were defined (Table 2).

**Table 2.** Definitions of levels of policies

Term	Definition
Health Policy	Decisions, plans and actions that are undertaken to achieve specific health care goals within a society. It defines a vision for the future which in turn helps to establish targets and points of reference for the short and medium term. Courses of action (and inaction) that affect the set of institutions, organizations, services and funding of the health system
Macrolevel policies	National high level policies that are generally broad in nature and require several inputs to achieve their aspiration. E.g. Reduce child mortality.
Mesolevel policies	National Programme level translation of a Macro policy into a working structure for an implementable programme. E.g. universal coverage of LLINs, targeted use of IRS
Strategy	Strategy is the direction in which the human and physical resources will be deployed and applied to achieve the objectives of the policies. E.g. Universal coverage of LLIN (the policy) through the free mass distribution to households (the strategy)

### Identification of key informants

The document review identified a broad range of stakeholders involved in the vector control policymaking process in Nigeria. These were categorized as: policymakers, researchers, private sector representatives, multilateral agency representatives and nongovernment organization (NGO) representatives. For the purposes of this study, policymakers include staff of the FMOH working in the National Malaria Control Programme (NMCP); NGOs include respondents from national NGOs implementing malaria control projects; multilaterals include United Nations technical agencies as well as multilateral funding institutions supporting malaria control; researchers, include those working in academia as well as those in national institutes of research; private sector refers to those in the commercial for-profit sector involved in the sale of vector control tools and insecticides. KIs were purposefully sampled to cover a comprehensive subset of the national stakeholders and represent each stakeholder category.

A list of KIs was drawn up and contacted to request interviews. A greater number of participants were interviewed from the NGO category as they made up the largest number and diversity of organizations and individuals contributing to the policymaking process. The initial list of KIs was expanded to include additional KIs identified during interviews.

All KIs were anonymized by assigning interviewee numbers so that their names and affiliations/institutions were not identifiable. However, quotes are assigned to their stakeholder category e.g. policymaker or NGO, in order to highlight their perspective.

### Data collection

The interviews followed a semistructured, open-ended format and was structured to explore the context, actors, process, content, power and the use of evidence in both (a) national vector control policy decisions and (b) in the planned scale-up of larviciding. See Supplementary File S1 for the interview guide. In March 2013, KT conducted the interviews in English in Abuja, Nigeria. The interviews were transcribed by a transcription service and KT checked all for accuracy.

### Data analysis

KT entered interviews into NVivo10 for data management and analysis. KT coded data according to the pre-defined themes in the policy framework using content analysis. Key themes were then summarized into areas of consensus and divergent views across stakeholder perspectives, and quotes used to illustrate key themes. All authors were involved in the analysis and interpretation of data.

## Results

A total of 14 national level stakeholders were interviewed: 3 policymakers, 1 researcher, 1 private sector representative, 4 multilateral agencies and 5 NGOs. The interviewees were a comprehensive subset of the potential respondents. All key in-country Roll Back Malaria Partners (RBM) and 14 of the 20 members of the Integrated Vector Management Subcommittee (IVM-SC) (the main technical body coordinating government and stakeholder input into vector control policy) were interviewed encompassing all identified stakeholder categories. The narrative for the results is based on the document review and KI perceptions. The normative process is as described by the Framework for the coordination of malaria control programme in Nigeria (National Malaria Control Programme 2009a) and the WHO Malaria Programme Review 2013 (WHO and Nigeria National Malaria Control Programme 2013) supplemented by the respondents’ perceptions of the ‘normal’policy process. The larviciding decision-making process is then compared and contrasted with this ‘norm’.

### Normative vector control policy analysis

#### Context

Nigeria is a Federation of 36 states, with three tiers of government (federal, state and local), each of which have a constitutional mandate to formulate and implement health policies and programmes (WHO and Nigeria National Malaria Control Programme 2013). The primary effect of the federal nature on the vector control policymaking context was the recognition by all respondents that, while the federal government has oversight of health policy, states can choose which vector control strategies to resource and implement based on their local context.

You see the nature of Nigeria is such that even when policies are made in the national level it is now left to the State to adopt it (Researcher)

Vector control policymaking is heavily influenced by WHO policies and recommendations of universal coverage of LLINs and the scale-up of IRS.

…We align A LOT with the Global Malaria Programme, WHO (Policymaker)

The health policy context is also influenced by the NMCP’s role in contributing to the wider national health, social and economic development objectives as articulated in the 2010 Nigerian National Strategic Health Development Plan (Federal Ministry of Health (Nigeria) 2010) and the Nigerian Vision 2020 strategy (Federal Government of Nigeria 2009). National policy documents revealed that national malaria vector control policymaking largely involves mesolevel policies and decisions around appropriate vector control strategies, i.e. the working structure of implementation. Thus, when KIs were asked about policymaking, they invariably spoke about strategy decision-making.

#### Actors

**Figure 2.**
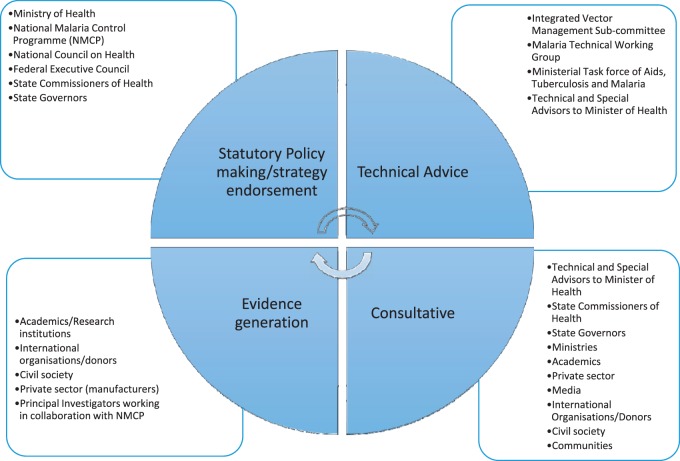
Functions of actors in vector control policymaking

Actors involved in vector control strategy decision-making generally participated in one or more of four main capacities: (a) policy/strategy decision-making; (b) advisory/technical; (c) consultative and (d) evidence generation. Figure 2 presents a synthesis of respondents’ views on the actors and their roles in the strategy decision-making process

### Policy/strategy decision-making

All KI’s recognized that the FMOH has ultimate responsibility for health policymaking in Nigeria. The NMCP as a department of FMOH executes policy, and fulfills a coordination role. States have concurrent jurisdiction to make policy and strategy decisions.

*National* malaria control program *is* statutorily *responsible for policymaking, as a division in the federal ministry of health because, you know, in Nigeria health is decentralized, national, state and then the local government levels”* (Policymaker).

Respondents cited the ministries of education, information, women’s affairs, environment, agriculture and finance as stakeholders in the vector control strategy decision-making process. Regulatory bodies such as the National Agency for Food and Drug Administration and Control (NAFDAC) who oversee the use of products such as insecticides were also cited as being critical to vector control strategy decision-making and implementation.

### Advisory/technical

All partners involved in malaria control in Nigeria are members of the Roll Back Malaria Partnership, led by the FMOH (WHO and Nigeria National Malaria Control Programme 2013). They provide advice to the NMCP, helping steer the overall direction of malaria control activities. As a group, they engage with the strategy decision-making process through the Ministerial Coordination Committee on AIDS, TB and Malaria (WHO and Nigeria National Malaria Control Programme 2013). The Ministerial Coordination Committee on AIDS, TB and Malaria is composed of three technical working groups (one for each disease). The Malaria technical working group has six sub committees (mirroring the six NMCP departments) including the IVM-SC. The main technical input into vector control strategy decisions by stakeholders is through the IVM-SC (National Malaria Control Programme 2009a).

### Consultative

KIs recognized that stakeholder consultation and consensus building is an integral part of the vector control strategy decision-making process. While there were no clearly defined junctions where consultations take place, it was recognized, across all respondent categories, that consensus should be built across a wide range of actors to facilitate strategy adoption and successful implementation.

It is recognised that malaria control is a collective responsibility and that in coming up with a strategy the platform for debate needs to be expanded to segments of the public, private, civil society. (NGO)

### Evidence generation

All KIs recognized that WHO recommendations provide the first line of evidence used to support or oppose a vector control strategy. However, it was also recognized that WHO recommendations are broad, leaving room for tailored interpretations at country level depending on local context.

…but we cannot just grab it (evidence) and change our policy…everything that comes into the country must be piloted, so the evidence we generate from that pilot will inform our decision as to whether we can include it in our policy. (Policymaker)

The NMCP coordinates with researchers from academic and research institutions to test new products in local trials for vector susceptibility and community acceptability. KIs from the public and private sector reported that the norm is for manufacturers to finance these trials with the NMCP and researchers overseeing the testing. This locally generated evidence is a prerequisite for the adoption of a vector control strategy, particularly in determining which insecticides to use.

Everything that comes into the country that has a potential of adding value into vector control must be piloted, so the evidence we generate from that pilot will inform our decision as to whether we can include it in our policy. (Policymaker)

Research institutions, such as the National Institute of Medical Research and individuals from a number of universities at national and in some cases at state level also participate in the IVM-SC. However, without a clearly formalized link or tradition of commissioning research by the NMCP this interaction is more opportunistic and based on personal relationships.

…The country as a whole does not have a health research plan and so when people do research they do research to publish, to get promotion……there is no formal channel, if I find something that is interesting the only thing I can do is talk to my director who can then call a press briefing. (Researcher)

#### Process

Interviewees from all categories reported that the normal policy process is initiated by a recognized failure in this strategy, the potential for new funding or the availability of new evidence. The IVM-SC is the forum for debating the need for, and evidence in relation to, a strategy adoption or change (National Malaria Control Programme 2009a). Interviewees all agreed that strategy is normally developed by the NMCP in collaboration with primarily the members of the IVM-SC, and channelled to the national coordinator and then the Minister of Health for endorsement with consultation of wider stakeholders at key points in the process.

These sub- committees are made up of partners who are experts…so when a policy is about to be made, these partners come together and brainstorm and take a decision on if that policy will benefit the country and if they think it would, then they work on it and then send it to the Honourable Minister of Health for him to ratify. (NGO)

In some instances, the decision is referred to the National Council on Health (NCH) and the Federal Executive Council (FEC). While there was uncertainty around what factors trigger the involvement of NCH and FEC, the use of government funding was cited as one potential factor.

A lot of time if that policy involves Nigeria’s money it will have to go to the FEC. (NGO)

#### Content

The policy targets and progress against them for malaria vector control in Nigeria are summarized in Table 3.

**Table 3.** Targets and progress: malaria vector control in Nigeria

Intervention	Progress to 2010 (National Population Commission and National Malaria Control Programme 2010)	2013 Target (National Malaria Control Programme 2009)
		
Indoor residual spraying	1% of target population received IRS	At least 80% of targeted population protected
Distribution of long lasting insecticidal nets	42% ITN household ownership and 29% use achieved	At least 80% of households with two or more LLINs/ITNs and 80% use by 2013
Larviciding	Piloted in four states	As appropriate in some selected areas

#### Evidence

When asked about evidence, KIs cited a wide range of sources as ‘trusted’ forms of evidence. These included WHO recommendations, results from household surveys, systematic reviews, meta-analyses, published literature, implementation research, feedback and results of locally generated evidence.

First and most important will be WHO recommendations, second will be published literature and documents from RBM working group, and then the last will be lessons learnt documentation and reports. (NGO)

KIs viewed scientific evidence as being useful for lobbying, creating awareness, documenting objective positions, defending decisions and catalyzing change. However, it was recognized that the necessary evidence was not always available. Furthermore, different stakeholder value and prioritize evidence differently. For example, respondents involved in funding malaria, typically external donors, appeared to place more value on the use of cost-effectiveness in decision-making.

… most of it is donor money, so donors are more aware of trying to get the best bang for their buck. (NGO)

Cost-effectiveness has been a concept of donors, UN agencies, partners and not government, sensu stricto. (Multilateral)

Whereas policymakers prioritize locally generated evidence.

What I am trying to say is that, local evidence is very critical, but you must compare it with the standard. (Policymaker)

Finally, it is recognized that in the process of decision-making, evidence can be ignored. As the debate proceeds from the technical to the political levels, wider political and socioeconomic factors can come more strongly into play.

But the disconnect is when it gets to minister of health a lot of political influence comes into play. (NGO)

#### Power

Interviewees identified two main groups of actors as having the most influence in the policy process. First, all stakeholder categories recognized the national and state government’s mandate to endorse policy decisions, thus conferring significant influence over the process.

For national policymaking, policy change decision-making, definitely as I told you before is the government. (Multilateral)

Second, donor influence was viewed as a key driver in the policy process. Respondents generally viewed the biggest catalyst for policy change as donor funding with one respondent citing the Global Fund to Fight AIDS, Tuberculosis and Malaria as an actor that has been able to utilize its financial power to drive through a number of policy changes.

‘The potential for new funding could drive a policy process, for instance if a donor has an interest in helping in the country changes its policy….And this is very common with Global Fund, for instance it has been able to drive a number of policy changes that go faster than ordinarily because the motivation to change the policy is there. (NGO)

### Larviciding policy analysis

#### Context

One of the key factors that facilitated the decision to scale-up larviciding was its potential to contribute to national economic development objectives through the technology transfer and the establishment of a microbial larvicide factory in Nigeria.

*In a country like Nigeria definitely there is interest to see more job creation, more wealth creation.* (NGO)

At the point at which an intervention is targeted at economic development but is said to have benefits for malaria control be it remotely or otherwise, and the audience for that has a bigger agenda and malaria control is just the smallest part of it, the tendency is that the malaria message gets drowned out. (NGO)

These views recognize that, contributions to the wider socioeconomic context can be highly influential in malaria vector control strategy decisions.

#### Actors

When asked about the actors involved in the decision to scale-up larviciding in Nigeria, interviewees cited the ECOWAS, the office of the Presidency of Nigeria and the Minister of Health. None of the interviewees mentioned that the decision had been technically debated at the IVM-SC level. A number of actors who normally participate in vector control policy decisions felt excluded from the larviciding decision, particularly those that play advisory, consultative and evidence generation roles.

‘It’s a closed (discussion)…in fact it’s not something we should talk about. That’s why the donor agencies or development partners in Nigeria are against that project, because it is shielded from them. (Private sector)

The discussion on larviciding did not include donors. (NGO)

#### Process

Interviewees reported that the decision-making process for larviciding deviated from the normal vector control decision-making process. The process flowed from the top (ECOWAS and presidential levels) to the bottom (NMCP level). The prevailing perception by all interviewees was that decisions were taken at high levels.

There is nothing people like us can do where the minister meets and ECOWAS takes a decision that this is what we want to do. (Researcher)

**Figure 3.**
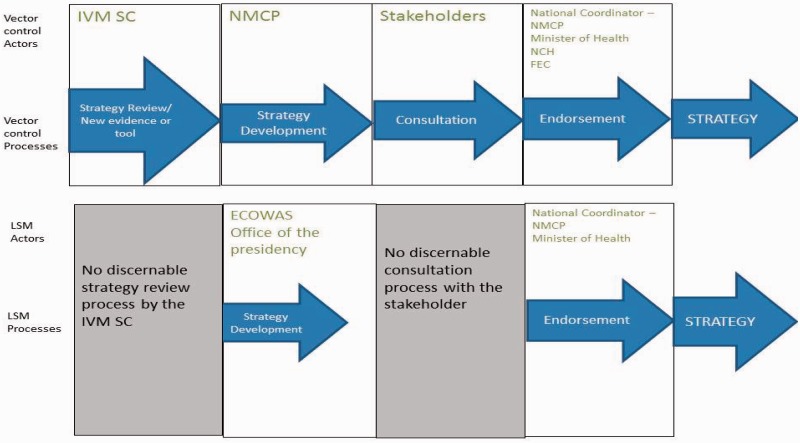
Vector control actors and processes: normative vs larviciding example

The normal vector control policy process is contrasted with the larviciding process in Figure 3. The larviciding decision process, as described by the respondents was shorter, appears to have been started by a decision at the highest levels of government and circumvented a number of policy processes and actors that are reflected in the normal processes of vector control policymaking.

#### Content

The Nigerian NMSP 2009–2013 currently recognizes the ‘limited application of larviciding and environmental management’ for vector control (National Malaria Control Programme 2009b). A new NMSP for 2014 and beyond is being developed and it is expected that it will feature larviciding more prominently to reflect the country’s commitment to nationwide scale-up. Beyond that, KIs were unable to give details of what the larviciding strategy would entail.

#### Evidence

Most respondents cited the use of some evidence to support the larviciding decision. KIs reported that ‘small’ evidence, i.e. evidence from the local pilot projects supported by the larvicide manufacturer, was used as the basis for the decision to implement larviciding at scale. This is in line with the reported norm for evidence in policymaking with locally produced evidence being used to validate international evidence in the local context. However, in this instance, results from local trials were used to support the use of the Bactivec strain which is not recommended by WHOPES and some actors, primarily those outside of government, perceived that the evidence produced was not open to scrutiny and debate.

It may not be big evidence, because I know people are looking for the big evidence,… we don’t have that type of evidence we are still generating. (Policymaker)

These studies were just to find out the efficacy of some of the larvicides, it is not an extensive one but just to determine the efficacy and once that has been determined, we said ok if we deploy this thing, following the appropriate standard and the best practices that will be able to achieve much hence we decided to do that. (Policymaker)

Members of the broader stakeholder group were either unaware of the role of evidence or questioned the quality of the evidence used in the decision-making.

I’m sure it (evidence) would have played some role, but then like I said, the decisions were taken at a higher level… (NGO)

I don’t want to use the word ‘questionable’. But also there are doubts, there are concerns as to the concrete, you know like the strength of their evidence. (NGO)

There is no evidence there. In fact, from what I know the matter has gone up high before the evidence were being gathered. (Private sector)

There was this larviciding project that was embarked upon by Rivers State government by

Labiofam where they used some insecticide and the report indicated that malaria prevalence in Rivers State actually had come down. (Policymaker)

The prevailing view amongst the wider stakeholders is that there exists little evidence and no policy framework to support nationwide larviciding in Nigeria with Bactivec. All stakeholders except for the policymakers held this view.

No scientific evidence to support the decision to carry out nationwide larviciding (NGO)

In Nigeria they got it wrong; the larviciding they want to do is not based on any policy. (Private sector)

The stakeholders’ objection to the larviciding strategy in Nigeria is summed up by three arguments. First, that Nigeria does not represent an appropriate context for the larviciding:

I think we do not represent the kind of place that larviciding would be effective on a large scale. (NGO)

However, the policymakers assert that the implementation of larviciding will be aligned to the WHO position on larviciding.

It is going to be in the context of that “few, fixed and findable,” unfortunately many people who are inside the box think that we are taking larviciding everywhere in Nigeria is not like that… no reasonable technical person will, it doesn’t make sense. *(*Policymaker*)*

Second, there were concerns that the selection of larvicide strain used was not WHOPES recommended, which contradicts the usual reliance on WHO recommendation and that the local evidence generated and used to support this decision was not sufficiently robust or independent.

Finally, and perhaps where the strength of the wider stakeholder’s argument lies is in the fact that larviciding represents a distraction from the primary malaria control interventions.

When you look at malaria control, spending all this money on larviciding when you don’t have sufficient funding to fill all your gaps for other commodities, you know, from a cost effective perspective, it would be more cost effective to take that money and put it into nets, if you’re doing vector control or RDTs or ACTs right, from a whole perspective of Malaria control. (NGO)

#### Power

The tripartite agreement, between ECOWAS, Venezuela and Cuba features financial and technical support to scale-up larviciding and technology transfer. Hence, financial power played a major role in the larviciding decision, but those exercising power were different to those perceived to wield this power in the normative situation.

Rivers State, the site of some of the pilot studies used as evidence for scaling-up larviciding, is the proposed site of the bio-larvicide factory (ECOWAS 2013). The Rivers State governor is a highly influential politician hence the technological and direct socioeconomic benefits of larviciding to Rivers State potentially created a formidable champion for scaling-up larviciding.

The commitment to scaling-up larviciding at the highest levels of government in Nigeria made the decision virtually unstoppable, with the hierarchical structure of the FMOH making the decision difficult to challenge.

At the point at which decisions are taken at the highest level of government the natural tendency from the government stand point is you support the decisions that are made by our superiors. *(*NGO)

In addition, there was an apparent restriction of information flow whereby all respondents, including those in NMCP, could not outline the details of the strategy for implementing larviciding, beyond the fact that it will be scaled-up nationwide. This control of information limits the policy actor’s ability to debate and build consensus around the intervention in the usual way.

## Discussion

This study is the first time that the decision to scale-up larviciding has been compared with normal policymaking processes in Nigeria. A review of the health policy analysis literature up to 2007 (Gilson and Raphaely 2008) included only six articles on malaria, all of which focused on treatment policies in Africa. Since then there has been a number of policy analyses in Sub-Saharan Africa looking at malaria treatment policy (Diap *et al*. 2010; Malisa *et al*. 2011; Martins *et al*. 2013; Nabyonga-Orem *et al.* 2014a,b), malaria in pregnancy interventions (Hill *et al*. 2013), and diagnosis (Bastiaens *et al.* 2011).

Changing malaria policy is generally seen to be a complex process (Williams *et al.* 2004; Amin *et al.* 2007). For example, the adoption of LLINs as global and then national policy across sub-Saharan Africa was a lengthy process involving multiple studies to demonstrate efficacy, effectiveness, cost-effectiveness and acceptability to end-users (Hill *et al.* 2006).

In the southern and east African contexts, policy analyses have been carried on integrated vector management (Mutero *et al.* 2012; Chanda *et al.* 2013), malaria control including vector control (Woelk *et al.* 2009; Mutero *et al.* 2014) malaria vector control (Cliff *et al.* 2010) and IRS (Montgomery *et al.* 2010). These studies have highlighted the value of local champions, international networks and the involvement of researchers in policy development in translating research into policy (Woelk *et al.* 2009). They also identified the critical importance of empirical data in informing decision-making and a need for a coordinated multipronged approach to vector control (Chanda *et al.* 2013). These studies demonstrate how factors such as outside influence and past experience of an intervention can slow the process of policy change (Cliff *et al.* 2010).

Policy analysis literature from South East Asia identifies similar critical factors in shaping policy despite being primarily focused on HIV/AIDS, and universal health coverage (Tangcharoensathien *et al.* 2004; Tantivess and Walt 2008; Teerawattananon and Russell 2008). Only one study in this context addresses malaria policy change but it focuses on the region’s unique epidemiological challenges. The focus on regional cooperation to deal with cross border malaria transmission and elimination is not currently directly comparable to the sub-Saharan African context (Bharati and Ganguly 2013).

The larviciding decision in Nigeria demonstrates a number of examples of power in policymaking. The decision was characterized by a top-down policy process with the FMOH overtly exercising its power to involve new actors and restrict the involvement of some traditional actors. All participants recognized that the Nigerian government had the ultimate decision-making power in policymaking. However, a tradition of involving the RBM partners, private sector, NGOs and the research community has created the expectation of wider participation and power sharing. This consultative process usually creates opportunities for debates to occur and promotes the production and exchange of evidence (Young 2005). Hence, the decision to restrict the actors involved and knowledge shared in the policy process allowed for selective use of evidence, akin to what Weiss describes as the ‘political’ use of research (Weiss 1979), causing concern over the quality of research evidence used in policymaking as observed in other contexts (Mutero *et al.* 2012). The actions of the FMOH undermined the norm of closely adhering to WHO policies, which traditionally set the context (agenda) for policymaking in malaria control.

Studies have cited a belief that donor preferences and agendas were exerting too much influence on malaria policies in the countries and that national level government actors are not adequately engaged in malaria control policymaking (Mutero *et al.* 2014). In this instance national leadership/ownership of a policy decision and engagement of different actors was highly controversial and heavily criticized. In 2012 WHO published an interim position statement on the role of larviciding in malaria control (World Health Organization 2012), in a bid to provide clear recommendations as a number of countries explored the use of larviciding. Alongside the WHO’s technical mandate, it is arguable that this statement had the power to influence global opinion i.e. an exercise of power as thought control. It is difficult to ascertain if the reaction of traditional actors was based only on the cited technical reasons, or if it was also due in part to displeasure at their power to influence being undermined. Either way this analysis highlights a potential conflict between greater national ownership of malaria policy decisions and adherence to internationally recognized standards and policy guidance which some view as an externally imposed donor construct.

This study demonstrates the persuasive power, especially to national policymakers of considering the wider socioeconomic context of vector control. The proposed local manufacture of the product, and the labour intensive nature of the intervention delivery, has potential to create large numbers of jobs and benefit the local and national economy. National level political actors may have selected the intervention based *inter alia* on the potential domestic economic benefits. The societal and economic benefits of controlling malaria are commonly used to justify intervention in malaria control. But when it comes to selecting between alternative interventions to control malaria, the process and actors tend to focus on evidence of health benefits (effectiveness) and cost-effectiveness. Cost-effectiveness analysis ignores the wider economic benefits of malaria control to domestic economies. Economic evaluations of alternative vector control interventions at country level would do well to consider the domestic economic impact of each approach and where these differ between interventions it should form the basis of discussion/debate with stakeholders beyond the malaria/health sector. If interventions are effective and can be shown to have a positive economic benefit (either directly, or indirectly through their impact on malaria) this could help generate additional domestic financing for malaria control. This would help achieve the Abuja declaration target of 15% government contribution to health expenditure (RBM 2000).

Political analysts recognize that the policymaking process is highly variable, ranging from a set of clearly defined stages followed by the rational weighing of competing options with the selection of the most optimal choice (Hogwood and Gunn 1984), to a process of ‘muddling’ through a complex and messy reality (Sabatier 2007). In this study, interviewees reported a clearly defined decision-making process where evidence is weighed and the most appropriate option implemented. The decision to switch from targeted to universal distribution of LLINs was cited as a particularly successful example. The larviciding decision is a deviation from the reported norm, arguably falling on the messy end of the policymaking spectrum. Stakeholders seeking to engage in the process need to be aware of the risk that, even in countries with rational policymaking systems, deviations from the established norm may occur and each decision can be different.

## Limitations

A number of potential limitations to this study exist. Firstly, as only 14 people were interviewed, inevitably some categories of stakeholders were underrepresented. Secondly, the study had limited access to what Shiffman terms ‘policy elites’, a recognized limitation of policy analysis at this level (Shiffman 2007). The interviewer’s inability to gain access to representatives from ECOWAS, the office of the presidency, and the Minister of Health, all who were identified as key actors in the decision to scale-up larviciding, but not identified in the desk review of ‘normal’ policy actors, denies the study a perspective that would have been valuable and enriching.

The decision to focus the study on perspectives at the national level may exclude valuable insight from the community level which may potentially support elements of the decision to scale-up larviciding.

The interviewer had spent time working closely with the NMCP and had a degree of ‘insider’ status, potentially allowing for greater insight into the policy analysis (Walt *et al.* 2008). In this instance, it allowed the interviewer increased access to respondents, the opportunity to investigate a ‘sensitive’ issue and an in-depth knowledge and understanding of the culture aiding in the interpretation of nonverbal cues.

## Conclusion

This study reaffirms that engaging powerful policy champions at the global and national levels can drive policy processes forward and thereby accelerate access to new vector control tools. It also suggests that a greater focus on the domestic economic benefits of malaria control could help generate greater domestic policy support and potentially finance for its control. However, care needs to be taken to ensure that inclusion of economic or other national goals does not result in health policies, which are not based on evidence of intervention effectiveness and internationally recognized standards of best practice.

## Supplementary Material

Supplementary Data
