# Supplementary material for: National malaria vector control policy: an analysis of the decision to scale-up larviciding in Nigeria
Source: Health Policy Plan. 2015 Jun 15;31(1):91–101. doi: 10.1093/heapol/czv055 (PMC4724167; doi:10.1093/heapol/czv055)
Supplement: Supplementary Data [file supp_31_1_91__index.html]

National malaria vector control policy: an analysis of the decision to scale-up larviciding in Nigeria — National malaria vector control policy: an analysis of the decision to scale-up larviciding in Nigeria — Supplementary Data 

# National malaria vector control policy: an analysis of the decision to scale-up larviciding in Nigeria

## Supplementary Data

files

- Supplementary Data - docx file
