# Supplementary material for: National malaria vector control policy: an analysis of the decision to scale-up larviciding in Nigeria
Source: Health Policy Plan. 2015 Jun 15;31(1):91–101. doi: 10.1093/heapol/czv055 (PMC4724167; doi:10.1093/heapol/czv055)
Supplement: Supplementary Data [file supp_czv055_SuppFile1_LarvicidingNigeria.docx]

**Stakeholder Interview Guide:** Maximising the translation potential of new malaria control tools through the application of health economics and policy analysis*.*

1. Name:
2. Gender:
3. Position:
4. Type of institution you work for?
5. Tell me a bit bout your background and the work you do in malaria control
6. What is your role in policymaking processes in malaria vector control?
7. **Vector control policy making in general**
   1. Can you describe to me the process by which national vector control policies are meant to be developed and agreed?
   2. Can you describe to me how you are engaged in the policy making process
   3. Do you see any barriers to things being taken up as policy?
   4. Which institutions/individuals play the biggest role and, in general who carries the most weight in influencing the decision making process?

Thinking now about the role of evidence in the policy making process…..

1. **The Role of evidence in the process**
   1. In what ways, if any, do you use the outputs of research to engage in the policy process?
   2. What kind of evidence do you find useful or most likely to influence policy?
   3. Aside from these sources of evidence, what other factors do you think come into play in decision about a new policy (e.g. donations from foreign governments, lobbying from interest groups (e.g. farmers), concerns about community acceptability/implementability etc). How?
   4. If you think there is an important research finding that needs to get into policy what institution would you speak to?
   5. What individuals/institutions ask you for information when they need to make decisions?

Thinking now about a recent change in policy relating to a malaria vector control tool ….

1. **Recent Malaria Vector Control Policy Change**
   1. Can we use the example of larviciding to discuss why policy changes?
   2. In your view, which of these factors played the most important role in
      1. (i) prompting the policy change discussion and
   3. Did the evidence for scientific research play and role and, if so, then in what way?
   4. Which institutions/individuals were instrumental/influential in the policy change process larviciding, ?
   5. Overall which three factors do you think have the greatest influence on decisions to change policy and adopt a new intervention strategy?

Thank you for your time and do you have any questions to ask me?
